# Sr Promoted Ni/W–Zr Catalysts for Highly Efficient CO_2_ Methanation: Unveiling the Role of Surface Basicity

**DOI:** 10.1021/acs.langmuir.3c02304

**Published:** 2023-11-29

**Authors:** Ahmed S. Al-Fatesh, Marie-Nour Kaydouh, Hamid Ahmed, Ahmed A. Ibrahim, Mohammed F. Alotibi, Ahmed I. Osman, Nissrine El Hassan

**Affiliations:** † Chemical Engineering Department, College of Engineering, King Saud University, P.O. Box 800, Riyadh 11421, Saudi Arabia; ‡ Petroleum Engineering Program, School of Engineering, Lebanese American University, P.O. Box 36, Byblos 1102-2801, Lebanon; § Institute of Refining and Petrochemicals Technologies, King Abdulaziz City for Science and Technology (KACST), P.O. Box 6086, Riyadh 11442, Saudi Arabia; ∥ School of Chemistry and Chemical Engineering, Queen’s University Belfast, Belfast BT9 5AG Northern Ireland, U.K.

## Abstract

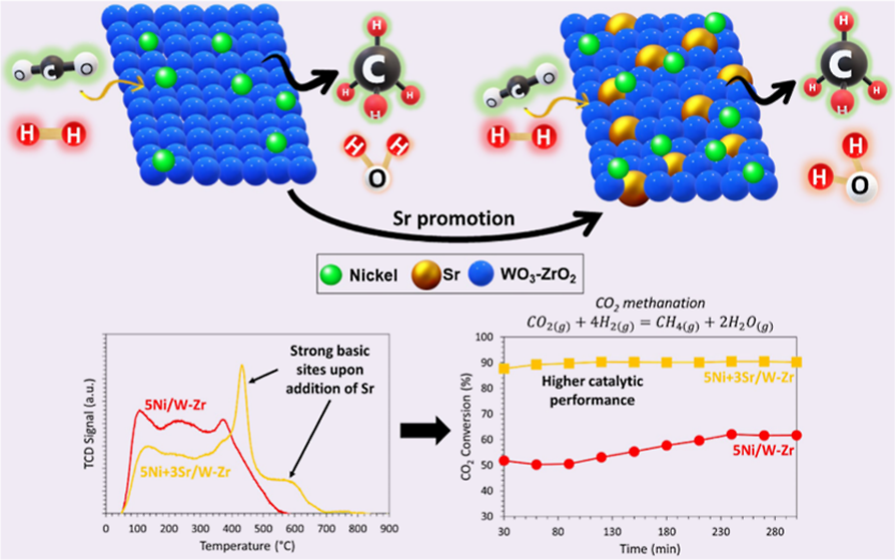

This study explores the employment of CO_2_ methanation for carbon dioxide utilization and global warming mitigation. For the first time, in this work, we combine the interesting properties of the WO_3_–ZrO_2_ support and the benefits of Sr to improve the performance of Ni-based catalysts in this reaction. Sr loading on 5Ni/W–Zr samples is increased to 3 wt %, resulting in improved surface basicity through strong basic site formation. After 300 min, the 5Ni + 3Sr/W–Zr catalyst exhibits high activity and stability, achieving 90% CO_2_ conversion and 82% CH_4_ yield compared to 62 and 57% on 5Ni/W–Zr. Limited sintering and absence of carbon deposits are confirmed by temperature-programmed oxidation, XRD, Raman, and TEM analyses at 350 °C for 300 min. Sr promotion creates additional CO_2_ adsorption and conversion sites, enhancing the catalytic performance.

## Introduction

The growth of the global population, coupled with the expansion of economic and industrial activities, has resulted in a significant accumulation of greenhouse gas emissions in the Earth’s atmosphere. Addressing this pressing issue by reducing anthropogenic CO_2_ emissions is of utmost importance to effectively mitigate the adverse effects of global climate change. It is essential to align our efforts with the objectives outlined in the Paris Agreement, which aim to limit the increase in global temperature to 2 °C and achieve the ambitious target of net zero carbon emissions by the year 2050.^1^ In this context, carbon dioxide holds immense potential as a valuable feedstock for producing useful chemicals and fuels through carbon capture and utilization (CCU). By harnessing CO_2_ as a resource rather than treating it solely as a waste product, we can drive the development of innovative technologies and catalytic systems that enable its conversion to high-value products. Embracing CCU not only presents an opportunity to reduce CO_2_ emissions but also offers a sustainable pathway toward a circular carbon economy, where CO_2_ is effectively recycled and utilized, thereby contributing to climate change mitigation.^2,3^ One viable approach for utilizing carbon dioxide is through its hydrogenation into methane, which serves as a substitute or synthetic natural gas.^4−6^ The process involved in converting CO_2_ and hydrogen into methane is commonly referred to as CO_2_ methanation, which can be regarded as a power-to-gas conversion.^7−9^

Furthermore, to align with the primary objective of clean energy production, it is essential to consider generating hydrogen coreactants from renewable sources rather than relying on fossil fuels. Methods such as water electrolysis or solar thermochemical water splitting offer promising pathways for renewable hydrogen generation.^10,11^ Water electrolysis employs electrical energy from renewable sources to split water molecules into hydrogen and oxygen, providing a sustainable and carbon-free source of hydrogen.^10^ Similarly, solar thermochemical water splitting utilizes concentrated solar energy to drive a chemical reaction that separates water into its constituent elements, yielding renewable hydrogen.^11^ By integrating these renewable hydrogen production methods with CO_2_ methanation, we can achieve a holistic and environmentally friendly approach toward clean energy production, ensuring a sustainable and low-carbon future. Consequently, CO_2_ methanation arises as an interesting power-to-gas approach that contributes to CO_2_ conversion and energy storage as well.^12−15^

From a thermodynamic standpoint, CO_2_ methanation is known as a highly exothermic reaction favorable at low temperatures (around 200–250 °C) and high pressures (ranging from 1 to 10 bar).^16,17^ Elevated temperatures can lead to the occurrence of the reverse water gas shift (RWGS) reaction, which can have an adverse effect on CO_2_ methanation by increasing the formation of carbon monoxide (CO) and reducing the production of methane (CH_4_).^18,19^ Operating at lower temperatures presents challenges as the CO_2_ methanation reaction becomes kinetically less favorable, necessitating the use of highly efficient catalysts to promote the hydrogenation of CO_2_ to CH_4_. While noble metals such as rhodium (Rh) and ruthenium (Ru) exhibit excellent activity and selectivity for methane production, their scarcity and high cost render them less economically viable for widespread commercial applications.^20,21^ As a result, cheaper Ni-based catalysts are being increasingly used as alternatives to the expensive Rh and Ru noble metal catalysts. These Ni-based catalysts have shown promising catalytic activity and selectivity for CO_2_ methanation.^22,23^ However, their catalytic performance is still facing challenges, particularly in terms of metal sintering and carbon deposition.^24^

Among the various catalyst systems that are studied and extensively reviewed,^7,8,20,25^ the utilization of zirconia (ZrO_2_) as a catalyst support has demonstrated notable advantages in terms of enhancing the reduction of nickel oxides and facilitating hydrogen spillover, which can effectively contribute to carbon elimination.^26−28^ These intriguing properties can be further fine-tuned and enhanced by incorporating dopants or modifiers into the support structure.^29^ The addition of reducible oxides, such as tungsten trioxide (WO_3_), to the catalyst support has been demonstrated to have several beneficial effects in CO_2_ methanation. One of the key advantages is the ability to stabilize the desired tetragonal t-ZrO_2_ structure,^30,31^ which is important for maintaining the structural integrity of the catalyst. By incorporating WO_3_, the aggregation and sintering of the support material can be effectively prevented.^32^ This leads to improved metal dispersion,^33^ ensuring a higher number of active sites available for the catalytic reaction. The enhanced metal dispersion contributes to better catalytic performance,^30^ facilitating the interaction between the active metal species and the reactants, promoting the desired chemical transformations. Additionally, the presence of WO_3_ in the catalyst support enhances the chemical and thermal stability of the catalyst system.^32^ This stability is crucial for maintaining the catalytic activity over extended periods of operation.^30−33^ Thus, the use of tungstated zirconia proved to be highly active in several applications, such as methane decomposition,^30^ isomerization,^31^ CO_2_ hydrogenation to methanol,^34^ and hydrogenolysis of glycerol.^33,35^

Recently, there has been growing interest in incorporating alkaline earth oxides into Ni-based catalysts. This approach has gained attention primarily due to the abundance and cost-effectiveness of alkaline earth elements, making them attractive alternatives for catalytic applications.^25,36,37^ Among these, strontium (Sr) proved to be an efficient promoter that can enhance metal dispersion,^38,39^ increase metal–support interaction,^40^ and boost the catalytic activity and stability by limiting carbon deposition^41,42^ in several applications such as dry reforming of methane,^39,40,42,43^ partial oxidation of methane,^41^ hydrogenation,^38^ and cracking reactions.^44^ In CO_2_ methanation, Sr was used as a promoter on Ni-based catalysts supported on Al_2_O_3_,^45−47^ CeZrO_*x*_,^48^ hydroxyapatite,^49^ and SiO_2_.^50^ The addition of 1, 2.5, 5, and 7.5 wt % Sr to Ni/Al_2_O_3_ resulted in a shift of the NiO reduction peak to 580, 620, 630, and 710 °C, respectively, suggesting an improvement of the metal–support interaction,^47^ as also observed on the CeZrO_*x*_ support.^48^ Sr promotion in Ni/Al_2_O_3_ catalysts enhances CO_2_ conversion from 45 to 80% (5% Sr–Ni/Al_2_O_3_) with negligible CO yield.^47^ Sr modifies the catalyst surface, promoting the activation of CO_2_ and selective methane production. This insight contributes to efficient CO_2_ utilization.^47^ In situ DRIFTS studies on the same catalysts^47^ demonstrated that the Sr-promotion of Ni/Al_2_O_3_ catalyst results in the formation of *CO and H_2_CO* intermediate surface species that can improve the CH_4_ yield. Studies also show that the addition of Sr improves the basicity of the catalyst, and this can favor the chemical adsorption of CO_2_ on the support.^48^ Guo and Lu^50^ revealed a promotional effect of Sr on Ni/SiO_2_ catalysts through the increase of CO_2_ conversion and CH_4_ selectivity from 64.7 and 97.5% for Ni/SiO_2_ catalysts up to 70.5 and 98.9% for Ni/Sr/SiO_2_ catalysts, respectively, at 350 °C and 15,000 mL h^–1^ g^–1^. Under these conditions, the Sr-promoted sample maintained high activity and remained stable for about 50 h while inhibiting Ni metal sintering.^50^

From this perspective, the addition of Sr shows promise in enhancing the catalytic performance in CO_2_ methanation. However, the combination of the WO_3_–ZrO_2_ support properties with Sr promotion in Ni-based catalysts for this reaction has not been investigated. This study aims to evaluate for the first time the effect of Sr addition to Ni/WO_3_–ZrO_2_ catalysts in methane production via CO_2_ hydrogenation.

## Experimental Section

### Materials

The mixed tungsten–zirconium oxide support (10WO_3_ + ZrO_2_) was provided by Daiichi Kigenso Kagaku Kogyo Co., Ltd., Osaka, Japan. The Ni and Sr precursors, nickel nitrate hexahydrate, Ni (NO_3_)_2_·6H_2_O, and strontium nitrate, Sr (NO_3_)_2_, of 98% grade were obtained from Alfa Aesar.

### Catalyst Preparation

All catalysts were prepared by using the wet impregnation method. The required amount of each precursor was first dissolved and mixed in 10 mL of distilled water. Then, the completing amount of the WO_3_ + ZrO_2_ support for 1 g was gradually added to the resulting solution, followed by heating and stirring until a slurry was obtained. After drying overnight in an oven at 120 °C, the samples were calcined at 600 °C with a 3 °C/min heating rate for 3 h. Finally, the catalysts were ground into powder and designated as 5Ni + *x*Sr/W–Zr, where *x* denotes the Sr loading (*x* = 0, 1, 2, and 3%). For all the samples, the Ni loading was fixed at 5 wt %.

### Catalyst Characterization

The N_2_ sorption isotherms were recorded on a Micromeritics Tristar II 3020 surface area and porosity analyzer after the samples were degassed at 200 °C for 3 h using N_2_. XRD data were collected using a Rigaku (Miniflex) diffractometer equipped with Cu Kα radiation and operated at 40 kV and 40 mA. The measurements were carried out for 2θ ranging from 5 to 90° at a step of 0.02°. Diffrac. EVA v4.2.1 software was used to evaluate the data. The obtained crystallite particle size was used to evaluate Ni metal dispersion using eq 1
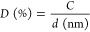
1where *C* is a constant equivalent to 97 for Ni and *d* is the crystallite diameter (nm) assuming spherical crystallites.^51^

TEM images of reduced and used catalysts were recorded on a transmission electron microscope JEOL JEM-2100F, USA, operated at 120 kV. These images were used to evaluate the particle size distribution and calculate the average diameter size. The Raman spectra of the used samples were registered using a laser Raman (NMR-4500) spectrometer (JASCO, Japan) with an excitation beam of 532 nm wavelength and an objective lens of 100× magnification. The laser intensity was set at 1.6 mW for a 10 s exposure time at three accumulations to protect the sample from being damaged by laser irradiation. Carbon deposition on the surface of the spent catalysts was evaluated by temperature-programmed oxidation (TPO) using a Micromeritics Auto Chem II 2920. The used catalyst was pretreated with argon for 30 min at 150 °C and then cooled to room temperature. The catalyst’s temperature was then increased from 50 to 1000 °C while being exposed to a 10% O_2_/He combination (flow rate of 40 mL/min). The integration of the resulting peaks provides the amount of oxygen consumed during the reaction, which is then used to calculate the amount of carbon deposited following the stoichiometry of the reaction (C_(s)_ + O_2(g)_ = CO_2(g)_). Utilizing Micromeritics Auto Chem II 2920, temperature-programmed desorption (TPD) was performed. 30 mL/min of a 10% CO_2_/He mixture was blasted over the catalyst at 50 °C for 30 min. Additionally, the sample’s temperature was increased up to 900 °C. At various temperatures, TCD finds the amount of CO_2_ that has been desorbed. The integration of the resulting peaks indicates the amount of CO_2_ desorbed, and the deconvolution of each peak is used to get the percentage of corresponding basic sites. The TPR measurements were performed on an AutoChem II apparatus 2920 (from Micromeritics). In detail, 0.07 g of each sample was first flushed with argon at 150 °C for 30 min and then cooled to room temperature. Next, the reduction was carried out using 40 mL/min of a H_2_/Ar mixture (1:9 vol %) by increasing the furnace temperature up to 900 °C (rate of 10 °C/min). Hydrogen consumption was recorded by using the thermal conductivity unit. The FTIR measurements were collected using a Nicolet Is-10 model (USA) Infrared spectrophotometer adopting the KBr technique. The samples were measured as KBr disks by mixing the sample with KBr (spectroscopic grade), where the solid samples were transferred to the cell after melting using an infrared lamp. The spectra of all the studied samples were measured under ambient conditions between 400 and 4000 cm^–1^ with a suitable scan resolution of 4 cm^–1^ and a scan rate of 16 cm/min.

### Catalyst Testing

To evaluate the catalytic performance of the 5Ni + *x*Sr/W–Zr series, 0.1 g of each sample was tested in a tubular fixed bed stainless-steel reactor (PID Eng. & Tech) having a length of 30 cm and an inner diameter of 9 mm. A thermocouple placed in the center of the catalytic bed is used to measure its temperature. Before testing, each sample was first activated in situ at atmospheric pressure by reduction under a hydrogen flow of 30 mL/min at 700 °C for 2 h. Then, the reactor was purged with nitrogen and cooled to 350 °C, and a reactive flow consisting of 16 mL/min H_2_, 4 mL/min CO_2_, and 10 mL/min N_2_ was introduced at an equivalent gas hourly space velocity (GHSV) of 18,000 mL/(h g_cat_). The methanation reaction was performed at atmospheric pressure at 350 °C for 300 min. The effluent gas was analyzed by online gas chromatography (Shimadzu 2014) using a TCD detector and two columns, Porapak Q and Molecular Sieve 5A.

The activity experiments were reproduced twice for all of the catalysts. To secure high measurement reliability and quality, the following approach was performed: in the catalytic test, nitrogen was used as an internal standard for the calculation of volumetric changes and concentrations of reactants and products. By using test gases that contain hydrogen, carbon dioxide, methane, and nitrogen in different concentrations, the GC response factors were determined to be in the range of 97–102% throughout all runs.

The conversion of CO_2_ and selectivity of CH_4_ were evaluated using eqs 2 and 3
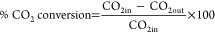
2
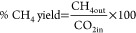
3

The catalytic performances of the samples were compared to thermodynamic data calculated using HSC 10 Chemistry software under identical reaction conditions. In detail, CO_2_, H_2_, and N_2_ were selected as inlets in the gaseous phase at a molar ratio of CO_2_/H_2_/N_2_ = 1:4:2.5, and the exiting products were composed of CH_4_, CO, and H_2_O, all in the gaseous phase. The equilibrium composition of the system was obtained at 350 °C and 1 atm, with and without considering solid carbon deposition of C(s) in the exiting stream.

## Results and Discussion

### Characterization of the Reduced Sample

**Figure 1:**
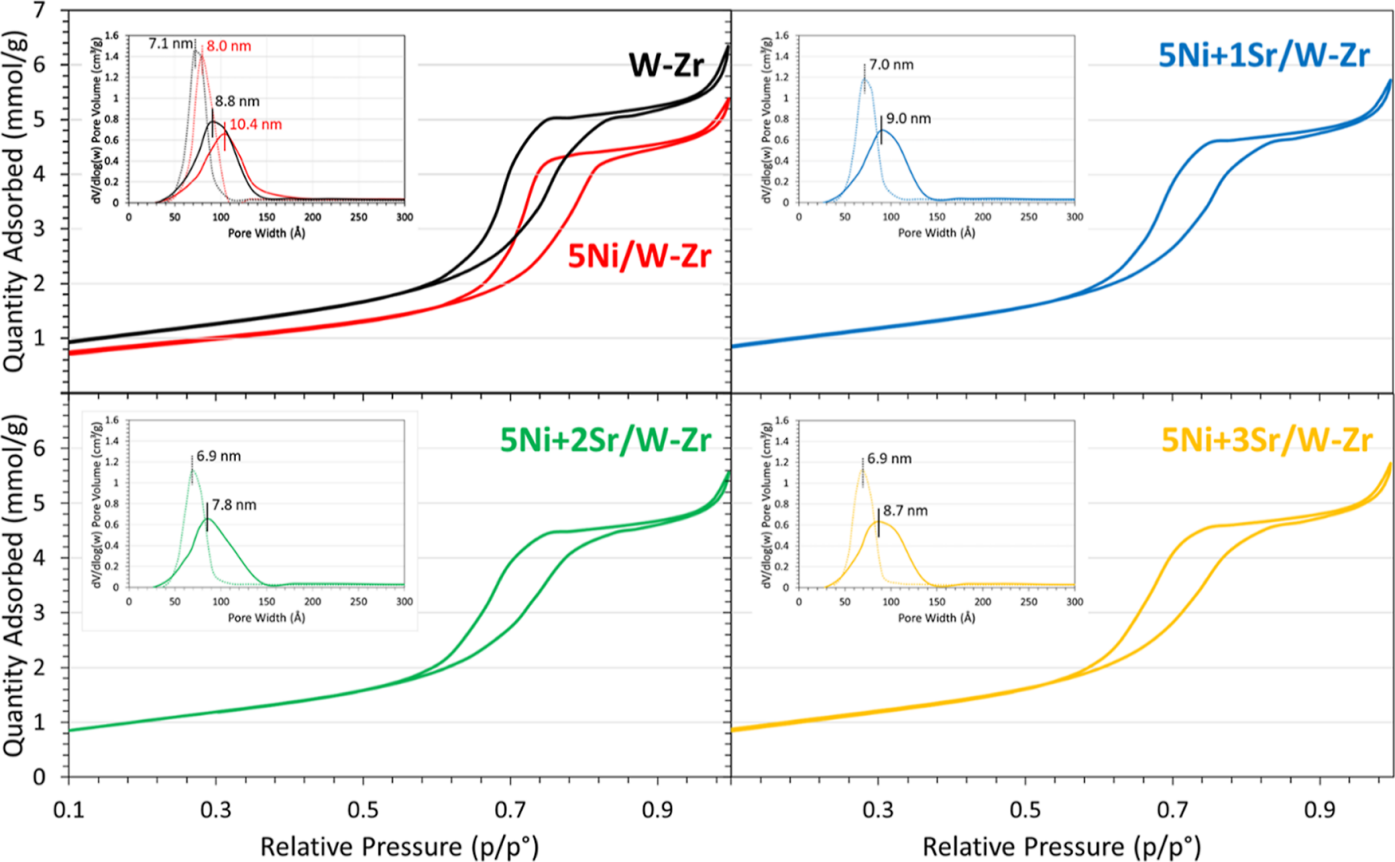
N_2_ sorption isotherms and pore size distribution (inset figures, solid line: adsorption, dashed line: desorption) for the reduced W–Zr support and 5Ni + *x*Sr/W–Zr (where *x* = 0–3 wt %) samples.

On Ni-based catalysts, it is commonly reported that Ni^0^ sites in their metallic state are the active sites for CO_2_ methanation.^52^ This is confirmed by the absence of any catalytic activity on blank tests using solely the bare supports,^45^ as will also be elaborated later in this work, validating the essential need of Ni^0^ active sites for the reaction. Indeed, metallic nickel plays an active role in the adsorption and dissociation of H_2_ into H atoms, while the constituents of the support influence the extent of CO_2_ adsorption.^20,23^ Consequently, it is primordial to reduce the catalyst before the methanation reaction in order to convert nickel oxide to metallic nickel. Thus, to align with the reduction step conducted prior to the methanation reaction test, the properties of the catalyst samples are evaluated in their reduced state. This section focuses on characterizing the reduced samples to gain insights into their properties. The reduced support and samples N_2_ sorption isotherms (Figure 1) correspond to a type IV isotherm with an H1 hysteresis loop that is typical of mesoporous materials with cylindrical mesopores that are uniformly sized. An unimodal pore size distribution is observed with pores ranging from 7 to 10 nm, depending on the catalyst. The addition of 5 wt % Ni to the W–Zr support decreases the BET surface area from 89.6 to 70.6 m^2^/g and slightly increases the pore size from 8.0 to 8.3 nm (Table 1). This can be explained by a possible expansion of the structure, as previously observed on comparable samples supported on yttria-zirconia.^53^ The addition of Sr along with 5 wt % Ni causes a decrease in the BET surface area to about 84 m^2^/g, indicative of pore filling by nickel and strontium. This is accompanied by a drop in pore volume and size from 0.25 cm^3^/g and 8.0 nm for the reduced W–Zr support to roughly 0.22 cm^3^/g and 7.4 nm for all Sr-containing samples, respectively (Table 1). Overall, regardless of the Sr loading, all Sr-containing catalysts show comparable textural properties.

**Table 1:** Textural Properties of the Reduced W–Zr Support and 5Ni + *x*Sr/W–Zr (Where *x* = 0–3 wt %) Catalysts

sample name	BET surface area (m^2^/g)	BJH adsorption cumulative pore volume (cm^3^/g)	BJH desorption cumulative pore volume (cm^3^/g)	BJH adsorption average pore size (nm)	BJH desorption average pore size (nm)
W–Zr	89.6	0.23	0.25	8.9	8.0
5Ni/W–Zr	70.6	0.19	0.21	9.6	8.3
5Ni + 1Sr/W–Zr	84.3	0.21	0.22	8.6	7.6
5Ni + 2Sr/W–Zr	84.1	0.20	0.22	8.3	7.4
5Ni + 3Sr/W–Zr	84.9	0.21	0.23	8.3	7.4

**Figure 2:**
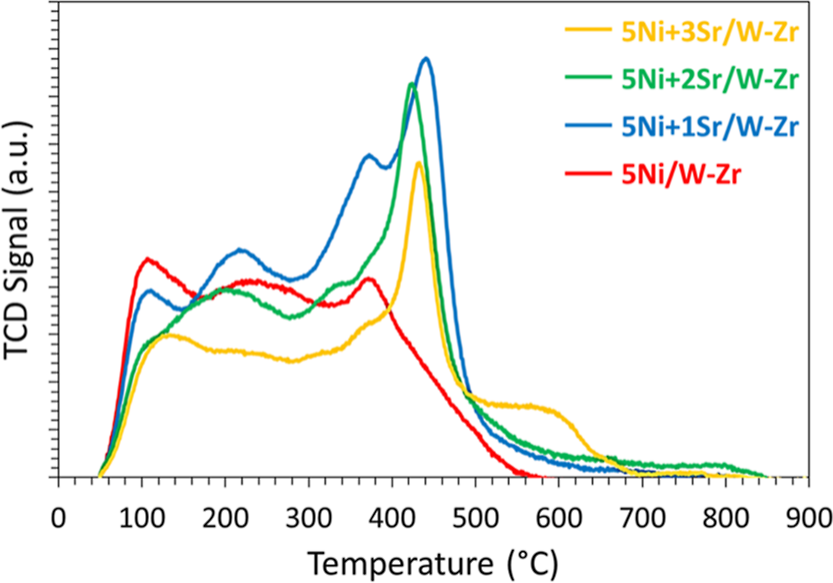
CO_2_-TPD results of reduced 5Ni + *x*Sr/W–Zr (where *x* = 0–3 wt %) catalysts.

The assessment of surface basicity is a crucial aspect that can be effectively carried out through the CO_2_-TPD. This technique provides valuable information about the distribution and strength of basic sites, contributing to a comprehensive understanding of the catalyst’s surface properties. It allows for identifying and quantifying different types of basic sites on the catalyst surface. Typically, peaks observed at low temperatures (50–200 °C) correspond to low basicity sites; those between 200 and 400 °C indicate moderate basicity, while peaks observed above 400 °C are associated with strong basicity sites.^45^ On the 5Ni/W–Zr reduced catalyst, low-intensity peaks appear at temperatures lower than 400 °C (Figure 2), indicating the presence of low and moderate basic sites on its surface. The addition of Sr to 5Ni/W–Zr results in an improvement of the surface basicity of the reduced samples since an intense peak is observed at around 450 °C for all Sr-containing samples (Figure 2). Furthermore, the 5Ni + 3Sr/W–Zr sample presents an additional peak at about 600 °C, suggesting the presence of very strong basic sites on its surface.^45^ In addition, the amount of CO_2_ desorbed during the CO_2_-TPD is also related to the Sr content. In numbers, the total CO_2_ amount desorbed increases from 0.040 mmol/g for 5Ni/W–Zr to 0.062, 0.096, and 0.101 mmol/g for 5Ni + 1Sr/W–Zr, 5Ni + 2Sr/W–Zr, and 5Ni + 3Sr/W–Zr, respectively. This shows the strong effect of Sr addition on the basicity of the samples and its high potential to increase the amount of CO_2_ adsorbed on the catalyst. Indeed, as the Sr content increases in the samples, the proportion of low basic sites is reduced, while that of strong basic sites is increased (Table 2). The abundance of strong basic sites upon adding Sr to 5Ni/W–Zr is analogous to the one reported upon adding 4% Sr to Ni/CeZr samples.^48^ Interestingly, such an increase in basicity was not observed in Ni/Al_2_O_3_ catalysts, even with the addition of up to 7.5 wt % Sr.^47^ This suggests that the type of support and its interaction with the metals play significant roles in influencing the basicity of the catalyst samples. The unique properties and interactions between the support material and metals can result in varying effects on the surface basicity, emphasizing the importance of considering the support and metal interactions in catalyst design and performance, as will be highlighted in the section related to the catalytic test results.

**Table 2:** CO_2_-TPD Data of Reduced 5Ni + *x*Sr/W–Zr (Where *x* = 0–3 wt %) Catalysts

sample	CO_2_ amount desorbed (mmol/g)	low basic sites (%)	moderate basic sites (%)	strong basic sites (%)	very strong basic sites (%)
5Ni/W–Zr	0.040	85	15	0	0
5Ni + 1Sr/W–Zr	0.062	44	4	53	0
5Ni + 2Sr/W–Zr	0.096	26	0	74	0
5Ni + 3Sr/W–Zr	0.101	36	0	56	8

**Figure 3:**
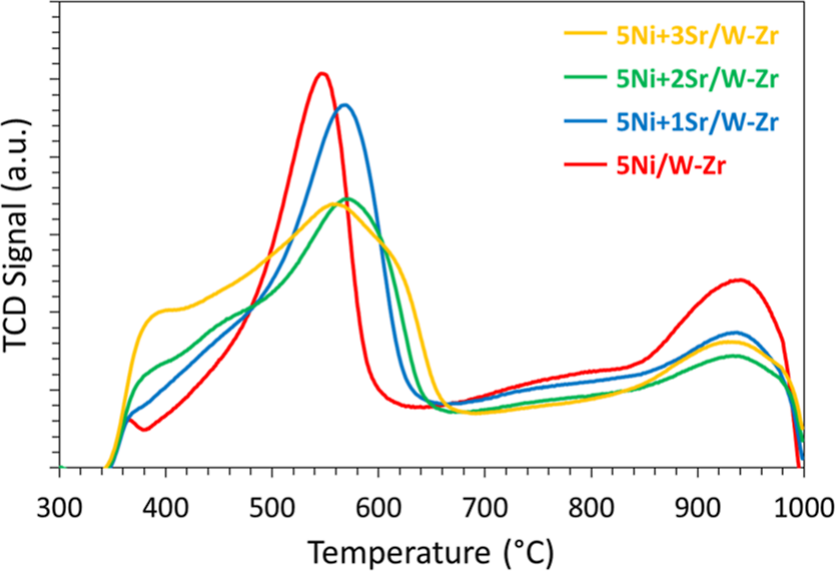
H_2_-TPR profiles of 5Ni + *x*Sr/W–Zr (where *x* = 0–3 wt %) catalysts.

The metal–support interaction can be further addressed using H_2_-TPR. The profiles of all samples show two major peaks (Figure 3), one centered at around 550 °C and another at 930 °C. However, the proportions of the two peaks are not identical for all samples (Table 3). As the Sr-content increases in the sample, the area of the first peak increases, while the area of the second decreases. In more detail, the increase in the Sr content from 1 up to 3 wt % results in an increase in the proportion of the first peak from 59 to 82%. This suggests that the addition of Sr results in lower metal–support interaction, which consequently improves and facilitates NiO reducibility since more NiO nanoparticles become reduced at lower temperatures.

**Table 3:** H_2_-TPR Data of 5Ni + *x*Sr/W–Zr (where *x* = 0–3 wt %) Catalysts

sample	H_2_ amount consumed (mmol/g)	area of 1st peak (%)	area of 2nd peak (%)
5Ni/W–Zr	1.98	48	52
5Ni + 1Sr/W–Zr	1.72	59	41
5Ni + 2Sr/W–Zr	1.43	65	35
5Ni + 3Sr/W–Zr	1.52	82	18

**Figure 4:**
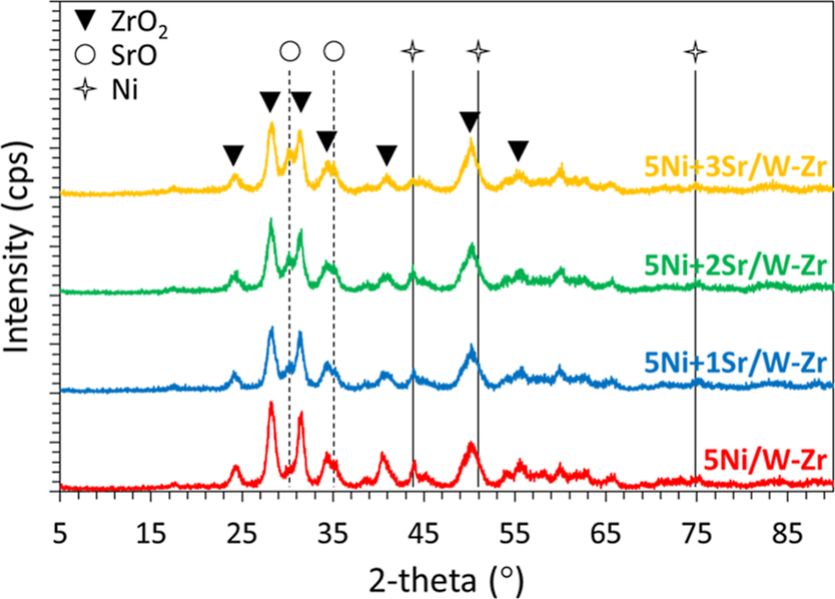
XRD patterns of the reduced 5Ni + *x*Sr/W–Zr (where *x* = 0–3 wt %) catalysts.

On the XRD data of the reduced catalysts (Figure 4), the diffraction peaks at 24, 28, 31.5, 34, 41, 50, and 55.5° correspond to ZrO_2_ (ref 00-065-0728), while those at around 44, 51, and 75° are attributable to metallic Ni^0^ (ref 01-077-9326) for all catalysts. On the calcined samples (data not shown), peaks characteristic of NiO were observed at 37 and 43°, similar to previous findings.^52^ The absence of these peaks on the reduced samples (Figure 4) confirms the complete reduction of NiO to metallic Ni^0^. The addition of Sr is detected by diffraction peaks at 30 and 35° that become more identified as the Sr loading is increased, characteristic of the partial reduction of SrO_2_ to SrO.^47^ Besides, the addition of Sr to 5Ni/W–Zr results in a decrease in the intensity of the Ni^0^ diffraction peaks, indicating a possible enhancement of Ni^0^ dispersion, as mentioned in the introduction and reported in the literature.^38,39^ While the major Ni^0^ peak at 44° is not well resolved, an estimation of the crystalline domain validated the decrease in size from 2.4 nm for 5Ni/W–Zr to 1.9 nm for 5Ni + 3Sr/W–Zr. Using eq 1, this decrease in the crystallite size translates into an improvement of Ni^0^ metal dispersion from 40% for 5Ni/W–Zr to 51% for 5Ni + 3Sr/W–Zr, validating the positive effect of Sr addition in enhancing dispersion.

### Catalytic Test Results

During CO_2_ methanation, the main reaction (eq 4) occurs, accompanied by several side reactions (eqs 5–7) that can either generate undesirable products such as CO or consume CH_4_, thus lowering the CH_4_ yield and selectivity.
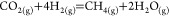
4
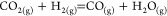
5
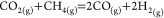
6
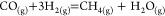
7

In addition, other side reactions (eqs 8–12) can also occur, resulting in undesirable solid carbon deposits on the surface of the catalysts.
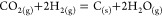
8
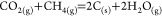
9
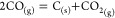
10
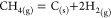
11
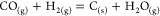
12

**Figure 5:**
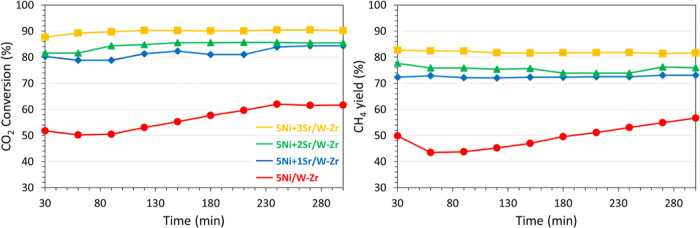
Catalytic test results of 5Ni + *x*Sr/W–Zr (where *x* = 0–3 wt %) catalysts. The methanation reaction was performed at atmospheric pressure at 350 °C for 300 min. The reactants’ flow rates were 16 mL/min H_2_, 4 mL/min CO_2_, and 10 mL/min N_2_ introduced at an equivalent GHSV of 18,000 mL/(h g_cat_).

The catalytic tests in this study were conducted at 350 °C for approximately 300 min. First, it is interesting to mention that the catalytic activity of a blank test using a WO_3_–ZrO_2_ support did not exceed 2% conversion, indicating the absence of any contribution from the catalytic support to the methanation reaction. According to the HSC calculations, the equilibrium conversion of CO_2_ at 350 °C under these operating conditions is estimated to be 89%, with a CH_4_ yield of 88%. However, on the 5Ni/W–Zr catalyst, the CO_2_ conversion remains stable at only 62%, while the CH_4_ yield gradually increases to 57% after 300 min on stream. These results highlight the need for the improved catalyst performance to achieve higher CO_2_ conversion and CH_4_ yield under the given operating conditions (Figure 5). The relatively low performance can be attributed to the limited prevalence of the primary methanation reaction (eq 4) and the potential simultaneous occurrence of the CO hydrogenation reaction, leading to CH_4_ production (eq 7). On the Sr-containing catalysts, after 300 min, the CO_2_ conversion varies following the Sr-loading as follows: 5Ni + 3Sr/W–Zr (90%) > 5N + 2Sr/W–Zr (85%) > 5Ni + 1Sr/W–Zr (84%). Similarly, the CH_4_ yield follows the same trend: 5Ni + 3Sr/W–Zr (82%) > 5Ni + 2Sr/W–Zr (76%) > 5Ni + 1Sr/W–Zr (73%). This order follows the same order obtained for the amount of CO_2_ desorbed during CO_2_-TPD (Table 2), as previously discussed. The inclusion of Sr in the catalysts demonstrates improved activity and stability compared with the Sr-free catalyst, indicating better catalytic performance. Additionally, increasing the Sr-loading enhances both CO_2_ conversion and CH_4_ yield. It is usually reported that the presence of medium basic sites facilitates the hydrogenation step and improves CO_2_ methanation.^54^ However, the data presented in this work show that the exceptional performance of the 5Ni + 3Sr/W–Zr catalyst can be rather attributed to the presence of highly strong basic sites, as validated by TPD analysis (Table 2). These sites facilitate CO_2_ adsorption and contribute to its higher conversion into CH_4_, thereby enhancing the overall catalytic efficiency. In addition, the ease of NiO reduction due to the low metal–support interaction in this case (Figure 3) can also be considered a favorable factor for the improved catalytic performance.

While several mechanisms have been proposed in the literature for CO_2_ methanation,^7,16,20,25,52,55−57^ the two widely accepted pathways include (i) the CO formation pathway where CO_2_ is first dissociated into CO-s and O-s, followed by the stepwise hydrogenation of CO-s to form CH_4_, and (ii) the formate formation pathway where adsorbed CO_2_ reacts with H-s to produce the HCOO-s formate intermediate which is then hydrogenated to CH_4_. Over Ni-based catalysts, the CO formation pathway proved to be more energetically favored.^58^

**Figure 6:**
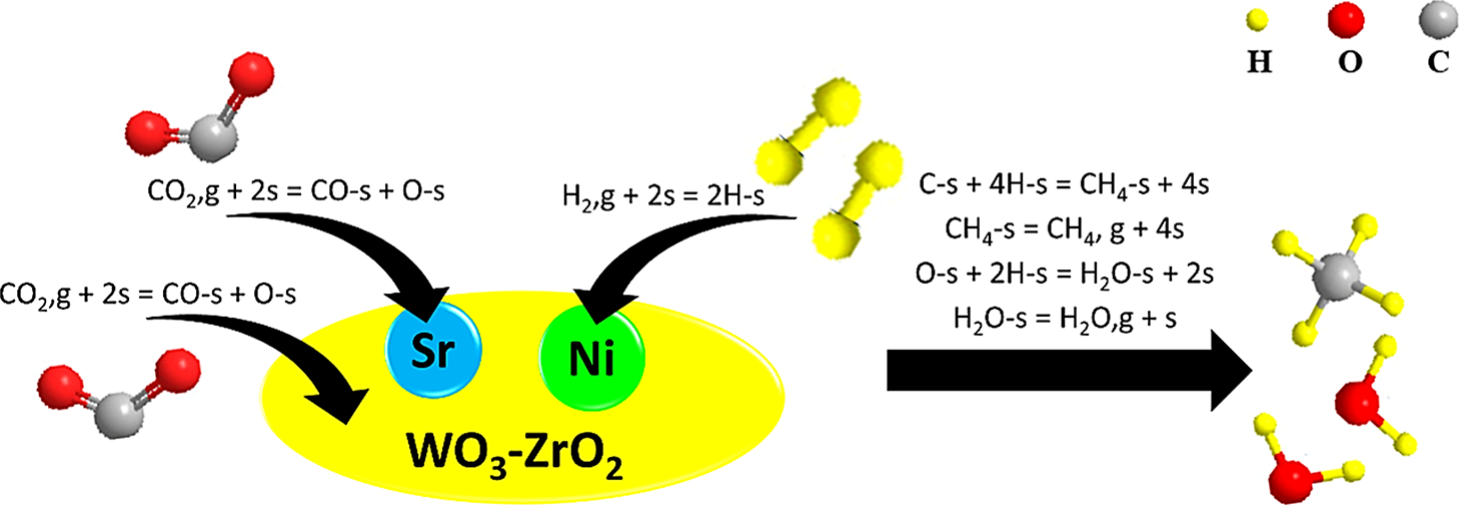
Schematic representation of the proposed reaction mechanism for CO_2_ methanation over 5Ni + *x*Sr/W–Zr catalysts.

In the current study, the high CO_2_ conversions and CH_4_ yields attained on the Sr-containing catalysts suggest that the main methanation reaction (eq 4) takes place on these samples with the limited occurrence of the side reactions (eqs 5–7). At 350 °C, the RWGS reaction (eq 5) and the dry reforming of methane (eq 6) are less favorable (Δ*G* = 15.8 and 82.3 kJ, respectively, compared to −50.8 kJ for eq 4 at 350 °C); thus, any CO produced as a side product is directly hydrogenated into CH_4_ and H_2_O (eq 7; Δ*G* = −66.6 kJ at 350 °C). This indicates that CO is not generated as a byproduct during the test, as confirmed by the high CH_4_ yield (Figure 5), but rather as a reaction intermediate. Consequently, the addition of Sr to the 5Ni/W–Zr catalyst results in an enhancement of the surface basicity, as confirmed by CO_2_-TPD (Figure 2), and contributes to the formation of additional sites for CO_2_ adsorption and dissociation into CO-s and O-s (Figure 6). This increases the formation of CO-s intermediates, whose subsequent dissociation into C-s and O-s is considered as the rate-determining step.^16^ Thus, on 5Ni + *x*Sr/W–Zr catalytic systems, the dissociation of hydrogen into H-s mainly occurs on the Ni sites, while the dissociation of CO_2_ into CO-s and O-s takes place on both the W–Zr support and the Sr sites, as illustrated in the suggested reaction mechanism (Figure 6). This boosts the activity of the catalysts and explains the superior performance of the Sr-containing samples compared to unpromoted 5Ni/W–Zr. In this regard, advanced in situ FTIR or DRIFT studies can be used to confirm this hypothesis and validate the proposed mechanism.

The catalytic performance of the catalysts shown herein is compared to that of similar Sr-containing Ni-based samples reported in the literature (Table 4). The catalysts mainly differ by the type of support used in each study, as well as by the Ni and Sr loadings. Despite containing the lowest amount of Ni, the catalysts investigated in this study exhibit noteworthy catalytic performance, particularly in terms of high CO_2_ conversion values at 350 °C. Notably, the catalysts, including the 5Ni + 3Sr/W–Zr catalyst, demonstrate superior behavior even when compared to catalysts with a higher Ni metal loading in the literature. This finding suggests that incorporating Sr and the specific catalyst compositions employed in this work contributes to enhanced catalytic activity, surpassing the performance of catalysts with higher Ni content. It was reported^50^ that a change in GHSV from 7,500 to 60,000 mL h^–1^ g^–1^ over Ni/SiO_2_ catalyst results in a notable decrease in CO_2_ conversion from approximately 78% to about 28% at 350 °C, respectively. This observation further emphasizes the significant impact of Sr addition to Ni catalysts supported on WO_3_–ZrO_2_, as reported in the present study. The incorporation of Sr enhances the catalytic performance and reinforces its effectiveness in promoting CO_2_ conversion, even under higher GHSV operating conditions.

**Table 4:** Comparison of Sr-Containing Ni-Based Catalysts in the Literature with the Current Work

sample name	support	Ni (wt %)	Sr (wt %)	catalyst weight (g)	H_2_/CO_2_ molar ratio	gas feed composition H_2_/CO_2_/N_2_	GHSV (mL/g h)	*P* (atm)	*X*(CO_2_) at 350°C (%)	*Y*(CH_4_) at 350°C (%)	ref
5Ni + 1Sr/W–Zr	WO_3_/ZrO_2_	5	1	0.1	4	4:1:2.5	18,000	1	84	73	this study
5Ni + 2Sr/W–Zr	WO_3_/ZrO_2_	5	2	0.1	4	4:1:2.5	18,000	1	85	76	this study
5Ni + 3Sr/W–Zr	WO_3_/ZrO_2_	5	3	0.1	4	4:1:2.5	18,000	1	90	82	this study
se-Ni/Sr/Si	SiO_2_	10	4	0.2	4	4:1:7.5	15,000	1	70.5	n.d.	(50)
1% Sr–Ni/Al_2_O_3_	Al_2_O_3_	20	1	0.5	4	4:1:1.3	11,400	1	60	55	(47)
2.5% Sr–Ni/Al_2_O_3_	Al_2_O_3_	20	2.5	0.5	4	4:1:1.3	11,400	1	75	75	(47)
5% Sr–Ni/Al_2_O_3_	Al_2_O_3_	20	5	0.5	4	4:1:1.3	11,400	1	80	80	(47)
7.5% Sr–Ni/Al_2_O_3_	Al_2_O_3_	20	7.5	0.5	4	4:1:1.3	11,400	1	75	75	(47)
Ni–Sr–Tb/Al_2_O_3_	Al_2_O_3_	25	5	0.1	3.5	n.d	9,000	1	58	n.d.	(46)
Ni/Sr-HAP	hydroxyapatite	10	n.d.	0.1	4	n.d.^a^	18,000	1	80	n.d.	(49)
Ni/Sr-HAP(F127)	hydroxyapatite	10	n.d.	0.1	4	n.d.^a^	18,000	1	83	82	(49)

a Ar balance.

### Characterization of Spent Catalysts

As mentioned earlier, following the CO formation pathway, adsorbed CO_2_ is dissociated into CO-s and O-s (Figure 6). Then, the resulting CO-s intermediates undergo dissociation into C-s and O-s. Consequently, carbon can accumulate on the catalyst if it is not continuously hydrogenated to CH_4_.

**Figure 7:**
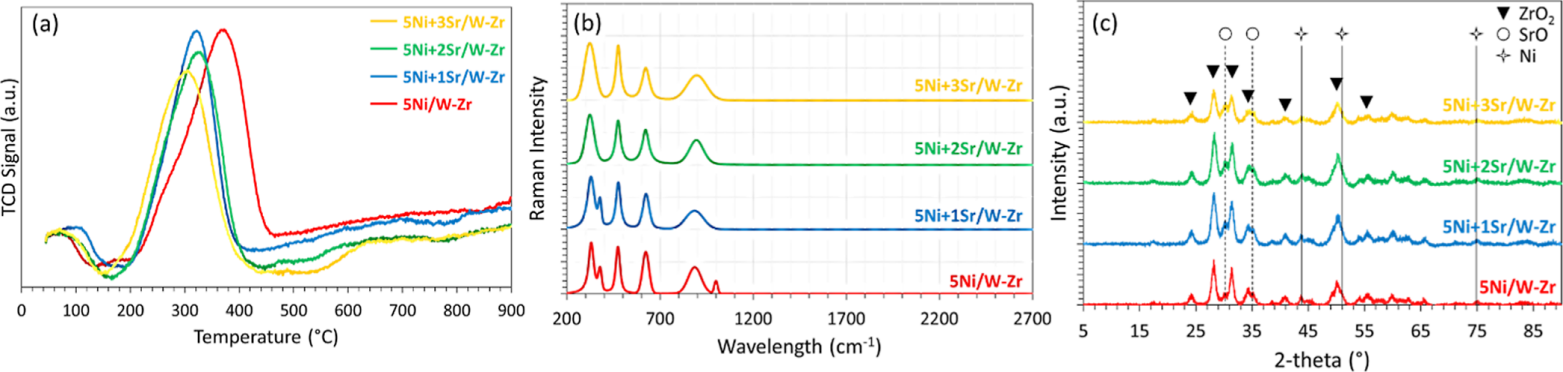
Characterization results of the spent 5Ni + *x*Sr/W–Zr (where *x* = 0–3 wt %) catalysts by (a) TPO, (b) Raman, and (c) XRD analyses.

To assess carbon deposition after 300 min of catalytic testing, TPO characterization was conducted on the spent samples. TPO analysis enables the identification of different forms of carbon. Peaks observed at temperatures below 250 °C indicate the presence of atomic carbon, while those between 200 and 600 °C signify amorphous carbon that can be readily gasified.^30^ Peaks at temperatures of >600 °C indicate the presence of graphitic filamentous carbon. This characterization technique provides valuable insights into the nature and amount of carbon deposits formed during the catalytic reaction.^30^ The results display a major peak between 200 and 450 °C (Figure 7a), implying the deposition of amorphous carbon on the surface of all samples,^30^ which is only an intermediate in the methanation reaction. Based on these TPO measurements, the amount of carbon observed on the samples is comparable and varies from 0.37 mmol/g for 5Ni/W–Zr to 0.27, 0.35, and 0.31 mmol/g for 5Ni + 1Sr/W–Zr, 5Ni + 2Sr/W–Zr, and 5Ni + 3Sr/W–Zr, respectively. However, the maximum of the peak appears to be related to the Sr-loading: the peak of 5Ni/W–Zr is centered at 370 °C, while it is shifted to lower temperatures of 330 °C for both 5Ni + 1Sr/W–Zr and 5Ni + 2Sr/W–Zr and to an even lower temperature of 310 °C for 5Ni + 3Sr/W–Zr. This suggests that the carbon deposited on 5Ni + 3Sr/W–Zr is the easiest to gasify, which further explains the high stability of this catalytic system during the methanation reaction.

**Figure 8:**
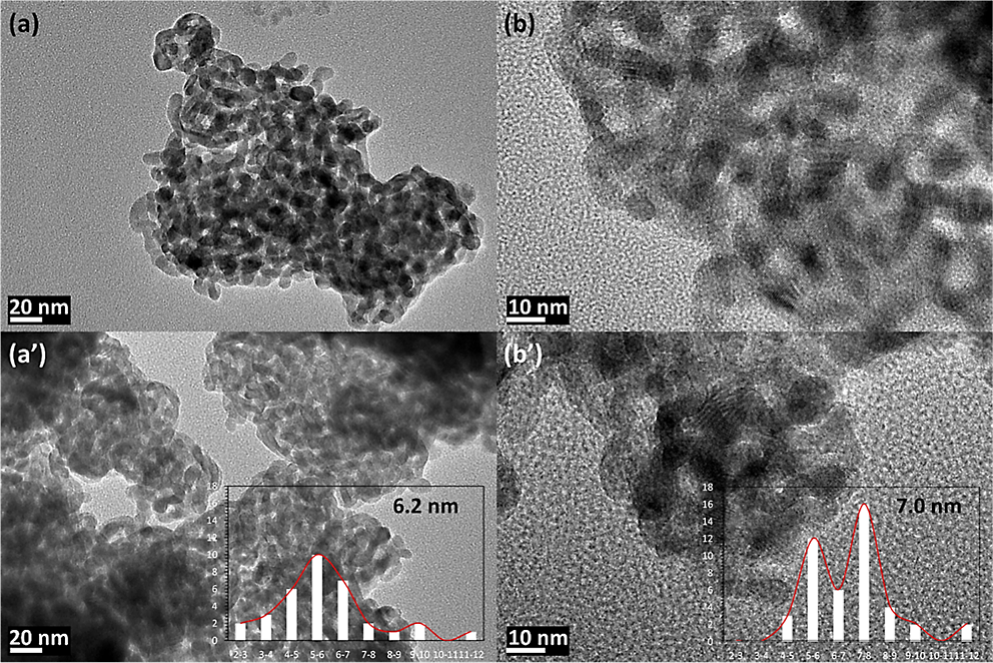
TEM images of the (a,b) reduced and (a’,b’) spent 5Ni + 3Sr/W–Zr catalyst.

On the Raman spectra (Figure 7b), the major peaks observed at 320, 470, 620, and 890 cm^–1^ are characteristics of the monoclinic ZrO_2_ support.^59−61^ The absence of the G-band at around 1580 cm^–1^, and the D-band near 1350 cm^–1^, validates the absence of carbon-containing materials, in agreement with the TPO results. Furthermore, the XRD peaks of the spent catalysts (Figure 7c) remained almost identical with those obtained on the reduced samples (Figure 4), suggesting the absence of important sintering in all samples. This idea is further supported by preserving a good metal dispersion and the absence of carbon deposition on the TEM images (Figure 8) of the 5Ni + 3Sr/W–Zr catalyst before and after catalytic testing.

## Conclusions

This study presents the characterization and testing of 5Ni + *x*Sr/W–Zr catalysts (*x* = 0–3 wt %) in CO_2_ methanation at 350 °C. Despite similar textural properties, the addition of Sr enhances the surface basicity, leading to increased CO_2_ adsorption and improved catalytic activity and stability. The 5Ni + 3Sr/W–Zr catalyst exhibits 90% CO_2_ conversion and 82% CH_4_ yield, compared to 62 and 57%, respectively, for the Sr-free 5Ni/W–Zr catalyst. TPO, XRD, Raman, and TEM analyses confirm limited sintering and no solid carbon deposition on 5Ni + 3Sr/W–Zr. These findings highlight the potential of Sr as a promising promoter for achieving high catalytic performance in CO_2_ methanation at 350 °C in Ni/W–Zr systems.

## Supporting Information Available

Brief description of the FTIR results of reduced 5Ni + *x*Sr/W–Zr (where *x* = 0–3 wt %) catalysts and calcined W–Zr support; FTIR spectra of reduced 5Ni + *x*Sr/W–Zr (where *x* = 0–3 wt %) catalysts; and FTIR spectra of calcined W–Zr support (PDF)
