# Supplementary material for: Sr Promoted Ni/W–Zr Catalysts for Highly Efficient CO2 Methanation: Unveiling the Role of Surface Basicity
Source: Langmuir. 2023 Nov 29;39(49):17723–32. doi: 10.1021/acs.langmuir.3c02304 (PMC10720459; doi:10.1021/acs.langmuir.3c02304)
Supplement: Supplementary file 1 — la3c02304_si_001.pdf [file la3c02304_si_001.pdf]

Supporting Information

Sr Promoted Ni/W-Zr Catalysts for Highly Efficient CO_2 Methanation: Unveiling the Role of Surface Basicity

Ahmed S. Al-Fatesh[a], Marie-Nour Kaydouh[b], Hamid Ahmed[a], Ahmed A. Ibrahim[a], Mohammed F. Alotibi[c], Ahmed Osman[d]*, and Nissrine El Hassan[b]**

[a] Chemical Engineering Department, College of Engineering, King Saud University, P.O. Box 800, Riyadh 11421, Saudi Arabia

[b] Petroleum Engineering Program, School of Engineering, Lebanese American University, P.O. Box 36, Byblos, Lebanon

[c] Institute of Refining and Petrochemicals Technologies, King Abdulaziz City for Science and Technology (KACST), P.O. Box 6086, Riyadh 11442, Saudi Arabia

[d] School of Chemistry and Chemical Engineering, Queen's University Belfast, Belfast BT9 5AG, Northern Ireland, UK.

Emails: aosmanahmed01@qub.ac.uk; aalfatesh@ksu.edu.sa; mfalotaibi@ksacst.edu.sa; nissrine.elhassan@lau.edu.lb

Table of content

- Brief description of the FTIR results.
- Figure S1. FTIR spectra of reduced 5Ni+xSr/W-Zr (where x = 0 – 3 wt.%) catalysts.
- Figure S2. FTIR spectra of calcined W-Zr support.

The interaction between species and the presence of functional groups on the surface of the catalysts are given by Fourier transform infrared (FTIR) spectroscopy (Figure S3). The absorbance bands at 3430 cm^{-1} and 1630 cm^{-1} are attributed to the stretching and bending vibrations of –OH hydroxyl groups or adsorbed water on the surface of the catalysts, respectively.[1,2] These two bands were also observed on the calcined W-Zr support (Figure S4). The band at 2890 cm^{-1} corresponds to the –C–H stretch,[3] while that at 2350 cm^{-1} corresponds to CO_2 adsorption on catalysts.[4] The bands between 500 and 800 cm^{-1} are assigned to zirconia[4,5] or to W–O–W bridging mode,[1] whereas those between 400 and 500 cm^{-1} can be assigned to Ni–O stretching[6] and Ni–O–Ni bond.[7]

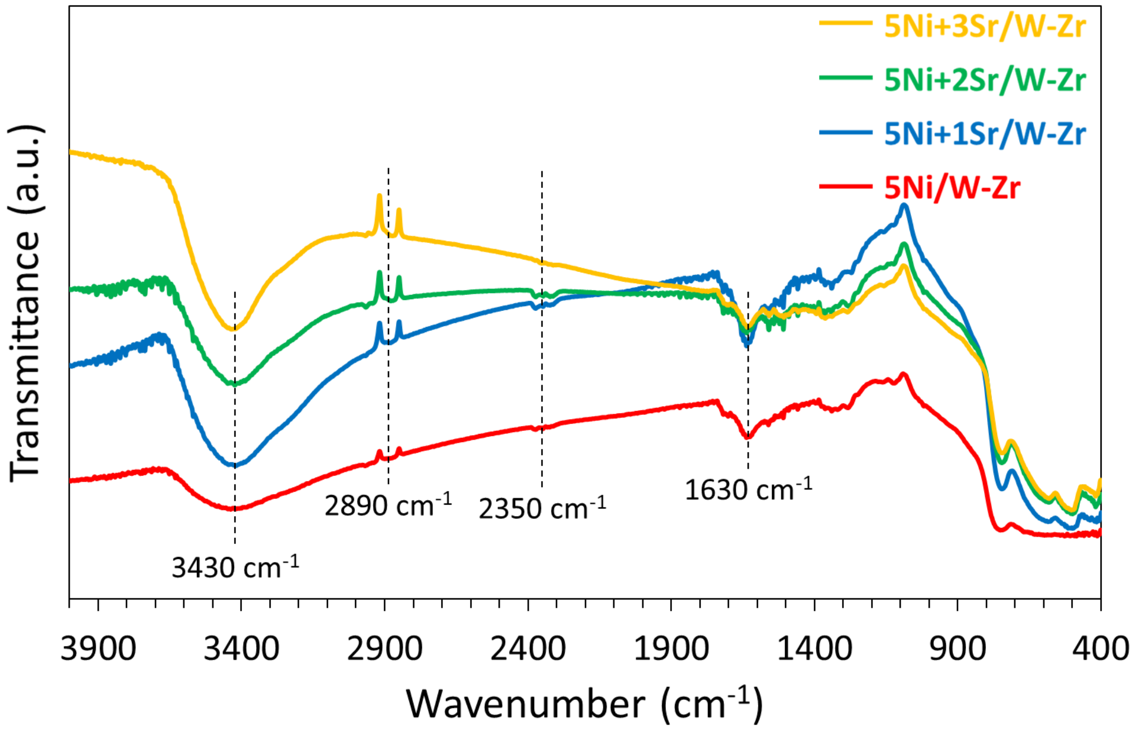

Figure S3. FTIR spectra of reduced 5Ni+xSr/W-Zr (where x = 0 – 3 wt.%) catalysts.

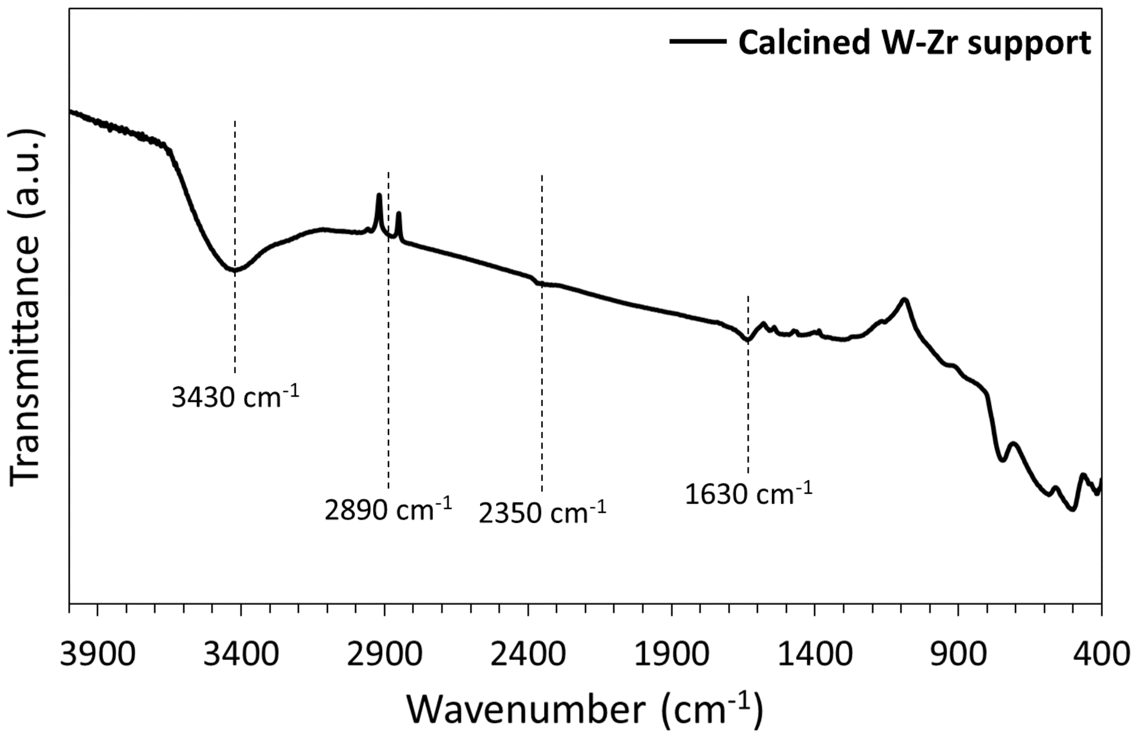

Figure S4. FTIR spectra of calcined W-Zr support.

REFERENCES

(1) Ramkumar, S.; Rajarajan, G. "A comparative study of humidity sensing and photocatalytic applications of pure and nickel (Ni)-doped WO3 thin films," *Appl. Phys. A,* vol. 123, p. 401, 2017.

(2) El-Salamony, R. A.; Al-Fatesh, A. S.; Acharya, K.; Abahussain, A. A. M.; Bagabas, A.; Kumar, N. S.; Ibrahim, A. A.; Khan, W. U.; Kumar, R. "Carbon Dioxide Valorization into Methane Using Samarium Oxide-Supported Monometallic and Bimetallic Catalysts," *Catalysts ,* vol. 13, p. 113, 2023.

(3) Namvar, F.; Hajizadeh-Oghaz, M.; Mahdi, M. A.; Ganduh, S. H.; Meshkani, F.; Salavati-Niasari, M. "The synthesis and characterization of Ni-M-Tb/Al2O3 (M: Mg, Ca, Sr and Ba) nanocatalysts prepared by different types of doping using the ultrasonic-assisted method to enhance CO2 methanation," *Int. J. Hydrogen Energy,* vol. 48, pp. 3862 - 3877, 2023.

(4) Kaur, M.; Pal, K. "Potential electrochemical hydrogen storage in nickel and cobalt nanoparticles-induced zirconia-graphene nanocomposite," *J Mater. Sci.: Mater. Electron. Vol.,* vol. 31, pp. 10903 - 10911, 2020.

(5) Mkhize, N. M.; Sithole, B. B.; Ntunka, M. G. "Heterogeneous Acid-Catalyzed Biodiesel Production from Crude Tall Oil: A Low-Grade and Less Expensive Feedstock," *J. Wood Chem. Technol.,* pp. 374 - 385, 2015.

(6) Budipramana, Y.; Taslim, E.; Kurniawan, F. "Synthesis nickel hydroxide by electrolysis at high voltage," *ARPN J. Eng. Appl. Sci.,* vol. 9, pp. 2074 - 2077, 2014.

(7) Parvas, M.; Haghighi, M.; Allahyari, S. "Catalytic wet air oxidation of phenol over ultrasound-assisted synthesized Ni/CeO2–ZrO2 nanocatalyst used in wastewater treatment," *Arabian J. Chem.,* vol. 12, pp. 1298 - 1307, 2019.
